# Nurse- and peer-led self-management programme for patients with an implantable cardioverter defibrillator; a feasibility study

**DOI:** 10.1186/1472-6955-6-6

**Published:** 2007-09-19

**Authors:** Esther STF Smeulders, Jolanda CM van Haastregt, Barbara K Dijkman-Domanska, Elisabeth FM van Hoef, Jacques ThM van Eijk, Gertrudis IJM Kempen

**Affiliations:** 1 Maastricht University, Faculty of Health, Medicine and Life sciences, School for Public Health and Primary Care, PO Box 616, 6200 MD Maastricht, The Netherlands; 2 University Hospital Maastricht, Department of Cardiology, PO Box 5800, 6202 AZ Maastricht, The Netherlands

## Abstract

**Background:**

The prevalence of cardiovascular disease is increasing. Improved treatment options increase survival after an acute myocardial infarction or sudden cardiac arrest, although patients often have difficulty adjusting and regaining control in daily life. In particular, patients who received an implantable cardioverter defibrillator (ICD) experience physical and psychological problems. Interventions to enhance perceived control and acceptance of the device are therefore necessary. This paper describes a small-scale study to explore the feasibility and the possible benefits of a structured nurse- and peer-led self-management programme ('Chronic Disease Self-Management Program' – CDSMP) among ICD patients.

**Methods:**

Ten male ICD patients (mean age = 65.5 years) participated in a group programme, consisting of six sessions, led by a team consisting of a nurse specialist and a patient with cardiovascular disease. Programme feasibility was evaluated among patients and leaders by measuring performance of the intervention according to protocol, attendance and adherence of the participating ICD patients, and patients' and leaders' opinions about the programme. In addition, before and directly after attending the intervention, programme benefits (e.g. perceived control, symptoms of anxiety and depression, and quality of life) were assessed.

**Results:**

The programme was conducted largely according to protocol. Eight patients attended at least four sessions, and adherence ranged from good to very good. On average, the patients reported to have benefited very much from the programme, which they gave an overall report mark of 8.4. The leaders considered the programme feasible as well. Furthermore, improvements were identified for general self-efficacy expectancies, symptoms of anxiety, physical functioning, social functioning, role limitations due to physical problems, and pain.

**Conclusion:**

This study suggests that a self-management programme led by a team consisting of a nurse specialist and a patient with cardiovascular disease seems feasible according to both patients and leaders. The programme may improve general self-efficacy expectancies, symptoms of anxiety, and quality of life (physical functioning, social functioning, role limitations due to physical problems, and pain) as well. Further investigation of the programme's effectiveness among a larger sample of ICD patients or other patient groups with cardiovascular disease, is recommended.

## Background

Worldwide, chronic conditions in general and specifically cardiovascular disease (CVD), are a major health burden [1]. The prevalence of CVD is increasing because of changed life expectancies, unhealthy lifestyles, and improved treatment options [2,3]. Because of these improved therapies, patients suffering from acute myocardial infarction have a considerably higher chance of survival. A substantial number of CVD patients receive an implantable cardioverter defibrillator (ICD), making it one of the most important recent advanced therapies in the prevention of sudden cardiac arrest due to life-threatening arrhythmias [4,5].

Adjusting to the consequences of CVD and regaining control in daily life can often be difficult, particularly after ICD implantation. Introducing a foreign body into the heart may be considered a major life event; ICD patients may be confronted with a changed body image, problems of psychosocial adaptation, and reduced quality of life [6]. In addition, symptoms of anxiety and depression after implantation appear to be common among ICD patients, affecting 24–87% and 24–33%, respectively, while 13–38% suffer clinically significant anxiety disorders [7]. So, although implantation may relieve much of the fear of sudden death, it also introduces new fears, so that the overall effect on psychological health is largely neutral [8]. Patients have to cope with the presence of a potentially life-threatening condition (i.e. ventricular arrhythmias) and the experience of potentially aversive treatment (i.e. shocks). According to Sears and colleagues [7], patients may increasingly limit their range of activities and inadvertently diminish their quality of life due to fear of shocks. To deal with the perceived lack of control, the fundamental psychological challenge for ICD patients is to derive perceived security from the ICD device [7,9]. Psychological interventions to enhance perceived control and acceptance of the device are therefore necessary [10]. A recent review showed that only a few studies assessed the effects of psychological interventions for ICD patients [11]. In the majority of these studies, no significant effects of these interventions were found, with exception of a cognitive behavioural therapy intervention [12]. In the majority of these studies, the lack of positive results may be explained partly by the small sample sizes and possibly also by the unstructured format of several of the interventions [11].

Structured self-management programmes have been developed by Lorig and colleagues since the early 1980s, including the widely disseminated 'Chronic Disease Self-Management Program' (CDSMP) [13-18]. The CDSMP is a generic group-based self-management programme, which is based on the principal assumption that patients with various chronic diseases can learn from each other as they have similar self-management problems and disease-related tasks [14]. The CDSMP, which is generally led by trained lay leaders, has been proved effective in several randomised trials with 6 to 24 months of follow-up [13,14,19]. It has been shown to significantly improve exercise level and communication with physicians, and to result in positive changes in health status and a reduction of health care utilisation among heterogeneous groups of patients with chronic conditions (including heart disease, lung disease, stroke, and arthritis) [14,15]. Although the CDSMP is originally developed for use in heterogeneous patient groups, we expect that the programme is also very well applicable in homogeneous patient groups such as ICD patients. The advantages of a homogeneous patient group may be that during the group process patients have better opportunities to share their disease specific problems, and are more likely to function as role models for each other. The CDSMP is based on Bandura's Social Cognitive Theory [20] and incorporates modelling, skills mastery, reinterpretation of symptoms, and social persuasion to enhance self-efficacy expectancies [13,15]. In cardiac samples, self-efficacy has been associated positively with life style changes and adherence to exercise programs [21]. In addition, the Social Cognitive Theory framework has been shown to be suitable for application to ICD patients improving physical and psychological functioning [22]. As in ICD patients the success of the device implantation may depend, in part, on the recipient's ability to adjust psychologically to the device [23,24], it is therefore important to assess the benefits of this self-management programme for ICD patients.

### Aim

This study presents the first exploration of the feasibility and the possible benefits of a Dutch nurse- and peer-led version of the structured CDSMP in a small group of ICD patients. To integrate the programme into the Dutch health care system, the Dutch version of the CDSMP incorporates a leader team made up of a cardiac nurse specialist ('professional leader') and a CVD patient ('peer leader'), instead of two lay leaders.

## Methods

### Design

A feasibility study with a one group pre-test/post-test design was carried out in order to assess the feasibility and the possible benefits of the programme.

### Participants

In 2003, a total of 95 patients received an ICD at University Hospital Maastricht. Of this group, 63 patients living in the three regions nearest the hospital were selected for our study to improve attendance at the programme sessions. Of these 63 patients, 37 persons were excluded due to participation in other studies or because they were no longer treated at University Hospital Maastricht. This procedure resulted in 26 eligible ICD patients who all received written information about the intervention and the study. Ten ICD patients were able and willing to participate in the study and attended the programme in the period May – June 2004.

### Intervention

The CDSMP consists of six weekly sessions, each lasting two and a half hours, and emphasizes the patients' central role and responsibility in managing their illness [13]. More specifically, patients learn three types of management tasks to achieve a positive and active management style: patients learn to deal with the medical management of the disease (medical management); to maintain, change and create new meaningful behaviours or life roles (role management), and third, to deal with the emotional consequences of having a chronic condition (emotional management). Regaining a sense of control by learning these three sets of tasks may improve quality of life in patients with a chronic disease [15,25] and perhaps also in CVD patients receiving ICD therapy.

Before the start of the study, the programme was translated into Dutch, including the book *"Living a healthy life with chronic conditions" *[26], written as a reference source for the material covered in the CDSMP. The patients received this book during the first programme session. Table 1 shows the CDSMP activities per session.

**Table 1:** Contents of the CDSMP

**Session 1**	
*Activity 1*	Introduction – Identifying common problems
*Activity 2*	Workshop overview and responsibilities
*Activity 3*	Differences between acute and chronic conditions
*Activity 4*	Introduction to cognitive symptom management
*Activity 5*	Introduction to action plans
*Activity 6*	Closing
**Session 2**	
*Activity 1*	Feedback/problem-solving session
*Activity 2*	Dealing with emotions (anger, fear, frustration)
*Activity 3*	Introduction to exercise
*Activity 4*	Making an action plan
*Activity 5*	Closing

**Session 3**	
*Activity 1*	Feedback/problem-solving session
*Activity 2*	Better breathing
*Activity 3*	Muscle relaxation
*Activity 4*	Fatigue management
*Activity 5*	Endurance exercise
*Activity 6*	Making an action plan
*Activity 7*	Closing

**Session 4**	
*Activity 1*	Feedback/problem-solving/making an action plan
*Activity 2*	Healthy eating
*Activity 3*	Distraction
*Activity 4*	Advance directives for health care
*Activity 5*	Communication skills
*Activity 6*	Problem-solving
*Activity 7*	Closing

**Session 5**	
*Activity 1*	Feedback/problem-solving/making an action plan
*Activity 2*	Medication usage
*Activity 3*	Making informed treatment decisions
*Activity 4*	Depression management
*Activity 5*	Self-talk
*Activity 6*	Guided imagery
*Activity 7*	Closing

**Session 6**	
*Activity 1*	Feedback/problem-solving
*Activity 2*	Informing the health care team
*Activity 3*	Working with your health care professional
*Activity 4*	Looking back and planning for the future
*Activity 5*	Closing

Although the CDSMP is originally led by two trained lay leaders, we decided to conduct the programme using a cardiac nurse specialist ('professional leader') and a patient with cardiovascular disease ('peer leader'). The choice for a nurse specialist was based on the fact that nurse specialists appear to play an important role in promoting self-care behaviour [27-30]. The CVD patient in the programme acts as a chronic disease role model. Furthermore, because of the exploratory approach of the study, several pairs of leaders were trained. They led the programme sessions in turns, in order to gain sufficient insight in the feasibility of the programme. Before the actual study started, three nurse specialists and four patients received four days of training on the CDSMP protocol. The leaders were trained by one of the researchers (author E.S.) and a cardiac nurse specialist from University Hospital Maastricht. Both had been instructed as master trainers at Stanford University.

### Data collection

#### Experiences with the programme

In order to gather information about the feasibility of the programme, four process outcomes were measured by structured evaluation forms: performance of the intervention according to protocol, attendance and adherence of the patients (i.e. patients' effort during the programme sessions), and patients' and leaders' opinions about the programme.

At the end of each session, the leaders described whether the activities during that session had been carried out according to protocol (i.e. the time limits for each activity adhered to, no activities skipped or shortened, or other training techniques used than required). In addition, the leaders recorded patients' attendance and adherence on a structured form. The overall level of patients' adherence with the programme activities was assessed by the leaders per session using a five-point scale (very good – very bad).

The patients' opinions about the programme were assessed after each session and directly after completion of the programme, using structured forms. The patients were asked to give their opinion about the relevance to their personal situation of each session (four-point scale; not at all relevant – very relevant) and to give a general report mark for the session (10-point scale; 1 to 10). Directly after completion of the programme, patients were asked to what extent they had benefited from it and which parts they had or had not appreciated. In addition, they were asked to assess the quality of the nurse specialist and the CVD patient as leaders (on a five-point scale from 'very good' to 'very bad'), to give overall report marks for the leaders and for the programme (10-point scales; 1 to 10), and to suggest possible improvements to the CDSMP. Experiences were assessed only of patients who attended at least three sessions. The leaders' opinion about the programme was assessed directly after completion of the six sessions by means of two structured group interviews: one for the professional leaders and one for the peer leaders. At these meetings, the leaders evaluated the protocol's feasibility and the cooperation with the co-leader.

#### Programme benefits

In order to assess the possible benefits of the programme, we measured self-efficacy expectancies, perceived control, symptoms of anxiety and depression, and quality of life at baseline and six weeks follow-up. Socio-demographic variables and co-morbidity were assessed once, at baseline.

Self-efficacy expectancies were measured with the 'General Self-Efficacy Scale' (GSES) [31]. Perceived control, or mastery, was measured by means of the Dutch version of the seven-item scale developed by Pearlin and Schooler [32]. Symptoms of anxiety and depression were measured with the Dutch version of the 'Hospital Anxiety and Depression Scale' (HADS) [33] which is considered to be unbiased by coexisting general medical conditions [34]. Health-related quality of life was assessed using eight of the nine subscales of the 'RAND 36-item Health Survey' (RAND-36) [35]: 'physical functioning', 'social functioning', 'role limitations (due to physical problems)', 'role limitations (due to emotional problems)', 'mental health', 'vitality', 'pain', and 'general health perception'. The psychometric properties of the Dutch versions of the GSES, perceived control scale, the HADS, and the RAND-36 were shown to be satisfactory in previous studies [34,36-39].

Socio-demographics (gender, age, marital state, living arrangement, educational level, and working situation) and co-morbidity (the presence of 19 chronic medical conditions) were assessed at baseline. Data were collected by self-administered questionnaires and telephone interviews. The telephone interviews were conducted independently by trained interviewers.

### Ethical considerations

The Medical Ethics Committee of Maastricht University/University Hospital Maastricht granted approval to conduct this study. All ICD patients were fully informed about the purpose and content of the study. Participation was voluntary and written consent was obtained prior to the measurement at baseline (May 2004). The investigation conformed with the principles outlined in the Declaration of Helsinki [40].

### Data analysis

The data with respect to the experiences with the programme were analysed in a descriptive way. To assess possible benefits for the ICD patients with respect to the outcome measures, means and standard deviations at baseline and at follow-up were computed. SPSS for Windows, version 12.0.1 was used.

## Results

### Patient characteristics

Patients' mean age was 65.5 years (SD = 7.9) and ranged from 50 to 76. All patients were male. The majority of patients did not live alone (n = 7), had a fairly high level of education (six patients had attended first stage of tertiary education) and were not employed (n = 8). The two patients with employment worked at least 32 hours per week. Mean time with the ICD was 8.3 months (range: 5–15 months) before inclusion in the study. The majority of the patients had a primary diagnosis of coronary artery disease (n = 8), of whom two patients had been resuscitated prior to ICD implantation because of ventricular arrhythmias. Patients' mean left ventricular ejection fraction was 32% (range: 20–53%), of whom the majority had suffered from at least one myocardial infarction prior to ICD implantation (n = 9) and experienced New York Heart Association (NYHA) Class II-III symptoms based on results of exercise tests (n = 8). All patients had received medical treatment for their heart condition in the preceding 12 months. With regard to shock history, two patients had experienced shocks in the period from implantation to inclusion in the study; one patient had experienced two shocks, the other patient had experienced an electrical storm of three shocks. Regarding co-morbidity, five patients reported no co-morbid chronic conditions, while the other five patients had received medical help in the preceding 12 months for at least one co-morbid chronic condition.

### Programme feasibility

#### Performance according to protocol

The leaders stated that each session was carried out largely according to protocol. Only minor protocol deviations were reported (i.e. spending more time on a topic than planned) as a result of problems with 'action planning' and 'problem solving' (first activity, session two and five). This was due to an imperfect match between the level of some of the action plans and the patients' daily activities. Also, the activities 'advance directives for health care' and 'communication skills' (session four) overran their time within the session, because these activities were appraised as difficult topics to deal with.

#### Patients' attendance and adherence

With regard to the attendance of the ten patients, the leaders reported that one person withdrew from the programme after the first session because the programme did not meet his expectations. One patient could only attend the first two sessions, because of hospitalisation for an ICD device problem. The eight remaining patients attended at least four sessions; one person could not attend the first two sessions because of interference with his work schedule, two persons were absent once due to other appointments, and five persons attended all sessions. The adherence per session and overall adherence was 'good' to 'very good', according to the leaders.

#### Patients' opinion about the CDSMP

The patients appraised the programme as 'rather relevant' to 'very relevant' and this opinion hardly differed for the individual sessions. The second and the last sessions were appreciated most by all patients. In the second session, all activities were appreciated equally: from 'feedback/problem-solving session', 'dealing with emotions', 'introduction to exercise', and 'making an action plan' to the opportunity to exchange personal experiences in that particular session. In the sixth session, 'working with your health care professional' was considered the most important activity, together with action planning in the activity 'looking back and planning for the future'. The report marks per session showed that patients' overall opinion about the programme was positive; the report marks ranged from 7.6 (first session) to 8.3 (second session).

Seven of the eight patients filled in the evaluation form at the end of the programme. On average, they reported to have 'benefited very much' from the programme and the overall report mark for the programme was 8.4. According to the patients, the best programme activities were: 'making an action plan' and 'problem-solving', cognitive symptom management techniques (e.g. 'better breathing' and 'muscle relaxation'), 'dealing with emotions', and 'advance directives for health care'/'making informed treatments decisions'. As regards the leaders, the ICD patients generally evaluated them as 'good', with the professional leader getting a higher score (report mark: 8.5) than the peer leader (report mark: 7.4). The fact that one of the leaders was a patient with cardiovascular disease had no added value for three patients. Finally, the patients made some suggestions for improvement of the CDSMP: three patients recommended discussing more ICD-specific subjects (e.g. ICD technology) or paying more attention to ICD-specific problems in daily life.

#### Leaders' opinion about the CDSMP

The leaders (n = 8) considered the programme to be feasible. Yet they sometimes found it difficult to carry out the programme exactly according to protocol (in particular, the activities 'making an action plan', 'problem-solving', and 'advance directives for health care'). Furthermore, the peer leaders experienced some role ambiguity and had some difficulties with modelling, which sometimes resulted in the professional leader interfering or taking over parts of the peer leader's activities.

### Programme benefits

As table 2 shows, the mean scores improved at follow-up, except for three subscales of the RAND-36 (vitality, general perceived health, and role limitations due to emotional problems). The largest differences in mean scores at baseline and at follow-up were found for general self-efficacy expectancies, symptoms of anxiety, and four RAND-36 subscales: physical functioning, social functioning, role limitations due to physical problems, and pain.

**Table 2:** Means and standard deviations at baseline and follow-up (n = 10)

	**At baseline**		**At follow-up (6 weeks)**	
	
	Mean	SD	Mean	SD
General self-efficacy expectancies* [16–80]	65.0	9.6	69.8	5.7
Perceived control* [7–35]	27.1	4.1	28.1	2.5
HADS** [0-21]				
*Symptoms of anxiety*	5.1	3.4	3.7	2.4
*Symptoms of depression*	3.9	3.7	2.9	3.1
RAND-36* [0–100]				
*Physical functioning*	66.0	17.4	72.8	22.5
*Social functioning*	75.0	22.5	84.7	25.6
*Role limitations (physical problem)*	50.0	48.4	69.4	42.9
*Role limitations (emotional problem)*	74.1	40.1	70.4	45.5
*Mental health*	79.6	13.6	82.7	13.9
*Vitality*	63.9	22.6	63.9	18.8
*Pain*	80.5	22.0	86.6	20.3
*General health perception*	56.7	19.5	56.7	22.9

* Higher scores indicate better functioning

** Higher scores indicate poorer functioning

Score between brackets refer to the theoretical range (underlined scores indicate most favourable scores)

## Discussion

From this feasibility study we learned that Lorig's structured self-management programme is applicable in a homogeneous group of ICD patients. The CDSMP was feasible according to patients and leaders. The patients appraised the programme as relevant and gave an overall report mark of 8.4. Furthermore, eight of the ten patients attended at least four sessions of the programme and only minor deviations from the protocol were reported. However, the leaders experienced some role ambiguity. This may be due to the fact that the CDSMP protocol does not include specific instructions for dividing the activities between the two leaders. Adding specific instructions in the protocol is therefore recommended, in order to diminish this experienced role ambiguity. Furthermore, becoming more experienced with the programme in practice may probably decrease role ambiguity as well. Next to the applicability of the programme, the CDSMP seemed to positively influence general self-efficacy expectancies, symptoms of anxiety, physical functioning, social functioning, role limitations due to physical problems, and pain. Although obviously our small-scale approach does not allow us to come to final conclusions regarding the effectiveness of the programme, our findings seem promising.

### Future research

This study presented the first exploration of the feasibility and the possible benefits of the CDSMP among ICD patients. Based on the results of this study, we recommend further investigation of the feasibility and effectiveness of the CDSMP among a larger sample of ICD patients or other CVD patient groups. Because of the exploratory approach of the study, several pairs of leaders led the programme sessions in turns, in order to gain sufficient insight in the feasibility of the programme. Obviously, it is recommended that in future projects each CDSMP class is led by one team of leaders to guarantee continuity of the programme. The turning pairs of leaders might have interfered at some level with the peer leaders' task to be a successful role model, as they could not optimally share information based on their own experiences with the patients in the group. However, to assure continuity of the process for the patients, after each session a structured report of that session was made by the professional leader to inform the other leaders about the group's progress. The fact that some of the patients considered the role of the CVD patient leader as of minor value for the group process might be explained by this discontinuity in leadership.

In the present study, the programme was led by a cardiac nurse specialist and a peer leader (i.e. a CVD patient), instead of two trained lay leaders. This adaptation was made in order to facilitate implementation in regular health care in the Netherlands and to control continuity of the programme if the peer leader should experience physical problems due to the disease. Both professional and peer leaders were positive about leading the sessions together. The professional leader could support the peer leader in conducting the programme, where the peer leader could discuss personal experiences with the disease during the sessions. Therefore, in future projects a combination of a professional leader and a peer leader per CDSMP class seems to be a good alternative to a team of two lay leaders. However, the final choice for the most appropriate composition of the teams is dependent on the setting in which it is going to be implemented.

Finally, in addition to assessing the effects of the programme among ICD patients, future studies could also study the effectiveness of the programme among partners of patients, as they experience physical and psychosocial problems as well in the caring process [41]. The CDSMP protocol allows partners of patients with chronic conditions to attend the programme, though for this feasibility study we decided not to invite partners for logistic reasons. In addition, future research should also assess whether the intervention positively influences health care utilisation in terms of visits to the outpatient clinic or hospitalisation days.

## Conclusion

The results of this study suggest that a group-based self-management programme led by a team consisting of a nurse specialist and a CVD patient is feasible for use among ICD patients. In summary, we may conclude that the intervention was generally performed according to protocol, that the patients' attendance and adherence were high, and that the opinion of both patients and leaders was positive. The experience of running the intervention can therefore be concerned as satisfactory. Furthermore, attending the CDSMP may positively influence general self-efficacy expectancies, symptoms of anxiety, physical functioning, social functioning, role limitations due to physical problems, and pain. As the small-scale approach of this study did not allow testing for statistical inferences, conclusions derived from this study should be taken with some caution. Therefore, we recommend assessing the effectiveness of the programme among a larger sample of ICD patients or other CVD patient groups in a randomised controlled trial.

## Competing interests

The author(s) declare that they have no competing interests.

## Authors' contributions

GK developed the project and obtained funding together with JvE and JvH. ES is the investigator and has worked together with EvH in developing the materials for this study, with input from the other authors. BD provided the list of patients for screening. ES drafted the manuscript, with input from the other authors. All authors read and approved the final manuscript.

## Acknowledgements

This research project was funded by the Netherlands Heart Foundation (2002B005) and the University Hospital Maastricht (PF 179), the Netherlands. This study was conducted within the School for Public Health and Primary Care of Maastricht University in the Netherlands in cooperation with Lorig and colleagues from Stanford University. We thank N. Steverink, H.A. Elzen, and J.P. Slaets from the University Hospital Groningen in the Netherlands for their willingness to share Dutch intervention materials. We want to thank Y.J. Stevenhagen for providing medical history information of the participants from the ICD database of the University Hospital Maastricht.

## Pre-publication history

The pre-publication history for this paper can be accessed here:



## References

[B1] World Health Organization Chronic conditions: the global burden. http://www.who.int/chronic_conditions/burden/en/.

[B2] Yach D, Hawkes C, Gould CL, Hofman KJ (2004). The global burden of chronic diseases: overcoming impediments to prevention and control. JAMA.

[B3] World Health Organization Why are Chronic Conditions Increasing?. http://www.who.int/chronic_conditions/why/en/index.html.

[B4] Kamphuis HC, de Leeuw JR, Derksen R, Hauer RN, Winnubst JA (2003). Implantable cardioverter defibrillator recipients: quality of life in recipients with and without ICD shock delivery: a prospective study. Europace.

[B5] Seidl K, Senges J (2003). Worldwide utilization of implantable cardioverter/defibrillators now and in the future. Card Electrophysiol Rev.

[B6] Duru F, Buchi S, Klaghofer R, Mattmann H, Sensky T, Buddeberg C, Candinas R (2001). How different from pacemaker patients are recipients of implantable cardioverter-defibrillators with respect to psychosocial adaptation, affective disorders, and quality of life?. Heart.

[B7] Sears SF, Todaro JF, Lewis TS, Sotile W, Conti JB (1999). Examining the psychosocial impact of implantable cardioverter defibrillators: a literature review. Clin Cardiol.

[B8] Lewin RJ, Frizelle DJ, Kaye GC (2001). A rehabilitative approach to patients with internal cardioverter-defibrillators. Heart.

[B9] Sears SF, Kovacs AH, Conti JB, Handberg E (2004). Expanding the scope of practice for cardiac rehabilitation: managing patients with implantable cardioverter defibrillators. J Cardiopulm Rehabil.

[B10] Sears SF, Conti JB (2002). Quality of life and psychological functioning of icd patients. Heart.

[B11] Edelman S, Lemon J, Kidman A (2003). Psychological therapies for recipients of implantable cardioverter defibrillators. Heart Lung.

[B12] Kohn CS, Petrucci RJ, Baessler C, Soto DM, Movsowitz C (2000). The effect of psychological intervention on patients' long-term adjustment to the ICD: a prospective study. Pace.

[B13] Lorig KR, Sobel DS, Ritter PL, Laurent D, Hobbs M (2001). Effect of a self-management program on patients with chronic disease. Eff Clin Pract.

[B14] Lorig KR, Sobel DS, Stewart AL, Brown BW, Bandura A, Ritter P, Gonzalez VM, Laurent DD, Holman HR (1999). Evidence suggesting that a chronic disease self-management program can improve health status while reducing hospitalization: a randomized trial. Med Care.

[B15] Lorig KR, Holman H (2003). Self-management education: history, definition, outcomes, and mechanisms. Ann Behav Med.

[B16] Fu D, Ding Y, McGowan P, Fu H (2006). Qualitative evaluation of Chronic Disease Self Management Program (CDSMP) in Shanghai. Patient Educ Couns.

[B17] Chan SC, Siu AM, Poon PK, Chan CC (2005). Chronic disease self-management program for Chinese patients: a preliminary multi-baseline study. Int J Rehabil Res.

[B18] Fu D, Fu H, McGowan P, Shen YE, Zhu L, Yang H, Mao J, Zhu S, Ding Y, Wei Z (2003). Implementation and quantitative evaluation of chronic disease self-management programme in Shanghai, China: randomized controlled trial. Bull World Health Organ.

[B19] Lorig KR, Ritter P, Stewart AL, Sobel DS, Brown BW, Bandura A, Gonzalez VM, Laurent DD, Holman HR (2001). Chronic disease self-management program: 2-year health status and health care utilization outcomes. Med Care.

[B20] Bandura A (1997). Self-efficacy: the exercise of control.

[B21] Dougherty CM, Johnson-Crowley NR, Lewis FM, Thompson EA (2001). Theoretical development of nursing interventions for sudden cardiac arrest survivors using social cognitive theory. ANS Adv Nurs Sci.

[B22] Dougherty CM, Thompson EA, Lewis FM (2005). Long-term outcomes of a telephone intervention after an ICD. Pace.

[B23] Beery T, Baas L (1996). Medical devices and attachment: holistic healing in the age of invasive technology. Issues Ment Health Nurs.

[B24] Beery TA, Baas LS, Matthews H, Burroughs J, Henthorn R (2005). Development of the implanted devices adjustment scale. Dimens Crit Care Nurs.

[B25] Lorig K, Funk SG, Tornquist EM, Leeman J, Miles MS and Harrell JS (2001). Self-management in chronic illness. Key aspects of preventing and managing chronic illness.

[B26] Lorig K, Holman H, Sobel D, Laurent D, Gonzalez V, Minor M (2000). Living a healthy life with chronic conditions: self-management of heart disease, arthritis, diabetes, asthma, bronchitis, emphysema and others.

[B27] Jaarsma T, Halfens R, Huijer Abu-Saad H, Dracup K, Gorgels T, van Ree J, Stappers J (1999). Effects of education and support on self-care and resource utilization in patients with heart failure. Eur Heart J.

[B28] Beswick AD, Rees K, West RR, Taylor FC, Burke M, Griebsch I, Taylor RS, Victory J, Brown J, Ebrahim S (2005). Improving uptake and adherence in cardiac rehabilitation: literature review. J Adv Nurs.

[B29] Sol BG, van der Bijl JJ, Banga JD, Visseren FL (2005). Vascular risk management through nurse-led self-management programs. J Vasc Nurs.

[B30] Frich LM (2003). Nursing interventions for patients with chronic conditions. J Adv Nurs.

[B31] Sherer M, Maddux JE, Mercandante B, Prentice-Dunn S, Jacobs B, Rogers RW (1982). The self-efficacy scale: construction and validation. Psychol Rep.

[B32] Pearlin LI, Schooler C (1978). The structure of coping. J Health Soc Behav.

[B33] Zigmond AS, Snaith RP (1983). The hospital anxiety and depression scale. Acta Psychiatr Scand.

[B34] Spinhoven P, Ormel J, Sloekers PP, Kempen GI, Speckens AE, Van Hemert AM (1997). A validation study of the Hospital Anxiety and Depression Scale (HADS) in different groups of Dutch subjects. Psychol Med.

[B35] Hays RD, Sherbourne CD, Mazel RM (1993). The RAND 36-Item Health Survey 1.0. Health Econ.

[B36] Bosscher RJ, Smit JH, Kempen GI (1997). Algemene competentieverwachtingen bij ouderen: Een onderzoek naar de psychometrische kenmerken van de Algemene Competentieschaal (ALCOS) [Global expectations of self-efficacy in the elderly: An investigation of psychometric characteristics of the General Self-Efficacy Scale]. Ned Tijdschr Psychol.

[B37] Kempen GI, Sanderman R, Miedema I, Meyboom-de Jong B, Ormel J (2000). Functional decline after congestive heart failure and acute myocardial infarction and the impact of psychological attributes. A prospective study. Qual Life Res.

[B38] Kempen GI (1992). Psychometric properties of GLAS baseline measures: a pilot study (in Dutch)..

[B39] van der Zee KI, Sanderman R (1993). Het meten van de algemene gezondheidstoestand met de RAND-36, een handleiding.

[B40] Rickham PP (1964). Human Experimentation. Code of Ethics of the World Medical Association. Declaration of Helsinki. Br Med J.

[B41] Albarran JW, Tagney J, James J (2004). Partners of ICD patients--an exploratory study of their experiences. Eur J Cardiovasc Nurs.

